# Effect of Lateral Wedge Insole With and Without Thigh and Abdominal Muscle Strengthening Exercises on the Balance of Young People With Genuvarum: A Randomize Controlled Trial

**DOI:** 10.1002/hsr2.70509

**Published:** 2025-03-18

**Authors:** Nafiseh Shahri, Aliyeh Daryabor, Mehdi Rezaei, Abbas Rahimi

**Affiliations:** ^1^ School of Rehabilitation, Shahid Beheshti University of Medical Sciences Tehran Iran; ^2^ Department of Orthotics and Prosthetics Physiotherapy Research Center, School of Rehabilitation, Shahid Beheshti University of Medical Sciences Tehran Iran

**Keywords:** balance, center of pressure, exercises, genuvarum, ground reaction force, lateral wedge

## Abstract

**Background and Aim:**

Genuvarum is a common lower limb deformity that affects the alignment of the leg in the frontal plane. This can lead to changes in the ground reaction force (GRF) and center of pressure (COP) of the foot, disrupting postural control strategies, and causing balance issues. This study aimed to determine the effect of strengthening exercises of thigh and abdominal muscles, along with use of a lateral wedge insole (LWI) on balance in young people with genuvarum.

**Methods:**

A total of 40 individuals aged 18–40 with genuvarum were split into two groups, with 20 people in each group. Participants in one group received only LWI (LWI group), while those in other group received LWI along with thigh and abdominal muscle strengthening exercises (LWI + exercises group). Balance tests were conducted before and after the 4‐week interventions under barefoot condition on a force plate. The tests included parameters related to COP in single‐ and two‐legged standing for static balance, and loading rate of F0 and F1 while walking, peak vertical GRF, and time to reach stability while jumping‐landing test for dynamic balance.

**Results:**

In the comparison between LWI + exercises group and LWI group, there was a significant decrease in variables related to COP during single‐leg standing and F1 loading rate during walking (*p* < 0.05).

**Conclusion:**

Incorporating muscle strengthening exercises targeting muscles around the thigh and abdominal region, in addition to using LWI, can be beneficial in individuals’ rehabilitation with genuvarum, helping to prevent injuries related to poor posture control.

**Trial Registration:** The current study was a randomized clinical trial, registered in the Iranian registry of clinical trials (registration number: IRCT20221103056387N1).

## 1 Introduction

Genuvarum is a common lower limb deformity in the frontal plane that can occur with age or in young people [1]. This condition alters the normal distribution of weight on the knee and ankle joints [2, 3], leading to misalignment of the body's mechanical axis and asymmetrical weight bearing. This, in turn, shifts the line of gravity in relation to the base of support, causing displacement of the ground reaction force (GRF) and the center of pressure (COP) of the foot during standing or walking. Consequently, postural control strategies are disrupted, affecting a person's balance and function [4]. Research indicates that genuvarum also impacts the activity of the thigh muscles. For instance, individuals with genuvarum may experience decreased activity in the gluteus medius muscle compared to those without the condition [4, 5]. Therefore, it is crucial to treat and rehabilitate individuals with genuvarum deformity to prevent complications and the progression of injuries.

Conservative treatments for genuvarum include surgery, knee braces, lateral wedge insole (LWI), and exercise therapy. The goal of these treatments is to correct the mechanical axis and alignment of the lower limb to correctly pass the line of gravity [5]. The LWI is considered an easier and more affordable way to reduce knee adductor moment in genuvarum [6]. Multiple studies have shown that the LWI increases pronation and valgus angle in the subtalar joint for people with medial knee osteoarthritis, which shifts the COP to the lateral and reduces the moment arm of the GRF, thereby reducing pressure on the medial knee and associated pain [6, 7, 8]. Additionally, evidence suggests that the LWI can reduce the average displacement and velocity of the foot COP. Furthermore, the LWI is found to improve somatosensory feedback by increasing foot contact with the surface and changing the distribution of pressure in the foot, ultimately leading to improved balance and reduced postural sway in the long term [9].

Genuvarum deformity can impact the body's balance due to defects in the muscular system functions. The interconnected nature of the muscular‐skeletal system means that a change in one part can affect other parts [10]. When walking during the single‐support phase, weakness in the thigh abductor muscles on one side can cause the pelvis to droop to the opposite side, moving the body's center of mass away from the knee and increasing the knee adductor moment. This weakness can lead to an increase in knee varus and the development of knee osteoarthritis [4, 11]. Evidence suggests that genuvarum places inappropriate stress on joints and disrupts body stability by altering the structure and function of muscles in lower limb joints and creating an imbalance in muscle strength [4, 12, 13]. Strengthening the hip abductor muscles can decrease the knee adductor moment, thus reducing knee varus. Therefore, improving muscle function can be an effective part of the treatment plan for individuals with genuvarum to enhance posture control.

In previous studies, two methods, namely, LWI and therapeutic exercises have been utilized to manage individuals with genuvarum. For therapeutic exercises, previous research has primarily focused on the muscles around the knee, such as the quadriceps, in this population [14, 15, 16]. Evidence indicates that strengthening the thigh muscles has led to reduced pain, improved physical symptoms, and better balance in individuals with knee osteoarthritis [13, 17]. However, there is a lack of studies on performing thigh strengthening exercises in people with genuvarum. For LWI, previous studies have mainly investigated its effect on reducing the varus moment of the knee [7, 8], but it is important to note that LWI can also impact balance in addition to its biomechanical and corrective effects. Therefore, the aim of this study was to compare the impact of a combination of passive (LWI) and active (strengthening exercises focusing on thigh and abdominal muscles) treatments versus passive treatment alone on the balance of individuals with genuvarum. The hypothesis was that exercises along with the use of LWI could improve static and dynamic balance outcomes more than LWI alone.

## 2 Methods

In this randomized controlled trial, 40 individuals with genuvarum between the ages of 18 and 40 participated as available samples. The inclusion criteria were apparently healthy individuals with genuvarum deformity, where the distance between the two medial knee epicondyles in the standing position was more than 3 cm, and the deformity was bilateral (although the two sides may not be the same size, the dominant side was considered). Exclusion criteria included the presence of other lower limb deformities such as femoral anteversion by Craig's test, history of corrective surgeries for the spine and lower limbs, use of assistive devices such as lower limb or spine orthoses, presence of any problems related to balance and coordination including cognitive, ear, and vision diseases, and a history of fractures and trauma in the last year.

The current study was a randomized clinical trial, registered in the Iranian registry of clinical trials (registration number: IRCT20221103056387N1), and the Ethics committee of Shahid Beheshti University of Medical Sciences approved all protocols (IR.SBMU.RETECH.REC.1401.149).

With *α* = 0.05, power = 90%, average values of *µ*_1_ = 6.02, *µ*_2_ = 5.24, standard deviations of *σ*_1_ = 0.7 and *σ*_2_ = 0.8 for the time to stabilization (TTS) variable from Mantashloo's et al. study (2018) (4), the sample size was calculated to be 20 people in each group using the following formula:

$$n=\left[\frac{{\left(z_{1-\alpha /2}+z_{1-\beta }\right)}^{2}(\sigma_{1}^{2}+\sigma_{2}^{2})}{{\left(\mu_{1}-\mu_{2}\right)}^{2}}\right].$$

### 2.1 Data Gathering Tool

To measure COP and GRF variables, we used a Bertec force plate made in the United States. The force plate has dimensions of 40 × 60 and is of model 4060‐08. It has a sampling frequency of 1000 Hz and is available in the biomechanics laboratory of the School of Rehabilitation at Shahid Beheshti University of Medical Sciences.

To make the insole, we used a foot scanning device called PT‐GAIT ANALYZER (F30L2000) from Paya Teb Fanavaran Company. The device has dimensions of 230 × 50 cm and a platform height of 1 cm. It is equipped with 11,440 resistive pressure sensors arranged in a matrix, providing a spatial resolution of 8 mm (1.4 sensors per square centimeter). The data sampling frequency is 100 Hz, with a hysteresis frequency of less than 3%. The pressure range of the device is from 0.5 to 100 pounds per square inch. The device extracts parameters including the force component in the vertical axis, pressure surface, and pressure amount, which were utilized in fabricating the insole.

### 2.2 Procedure

After visiting an orthotics and prosthetics clinic, individuals underwent an initial examination and then stood on a foot scanning device to record data on the pressure of their feet. This information was used to make custom insoles. Specifically, a 5 mm thick polyethylene insole with mild arch support and a lateral wedge was designed and produced using CNC software.

After all the participants received LWIs, they were divided into two groups. One group received only LWI, while the other group received LWI along with thigh and abdominal muscle strengthening exercises. Subsequently, all balance tests on a force plate were conducted with participants barefoot in a biomechanics laboratory. The data was obtained from the average of three tests in each condition, with participants resting for 1 min between each test condition [9].

Balance static defines as maintaining balance in unperturbed conditions, such as quiet standing or the ability to hold a base of support with minimal motion. To assess static balance, participants were instructed to stand on both legs on the force plate with their eyes open and maintain this position for 30 s. Subsequently, they were asked to stand on their dominant leg in a single leg position and maintain this stance for 30 s. The dominant limb is defined as the preferred limb used to hit a ball.

Dynamic balance refers to the ability to maintain postural control while moving, including during activities such as walking, turning or regaining balance on an unstable surface with minimal motion. Participants walked on the walkway at a self‐selected speed, ensuring that the dominant limb was placed on the force plane to evaluate the loading rate resulting from the vertical GRF. The GRF was normalized by dividing it by the subjects’ weight. F0 and F1 loading rate represent the slope of the vertical GRF as the transient force at initial contact and the first peak in the loading response phase, respectively.

For the jumping‐landing test, we first needed to calculate 50% of the subject's maximum jump height to standardize the vertical height of the jump [18]. The individuals stood sideways next to the wall, raised their hand, and we marked the highest point they reached. Then, they performed a vertical jump with full force, and we marked the point they touched with their hand. By comparing the mark from standing still and the average of three jumps, we obtained a number equal to 50% of the maximum vertical jump. After that, participants were instructed to stand barefoot behind a mark that was half the length of their lower limb from the center of the force plate. A thick bar on the force plate displayed the jump height equivalent to 50% of the individual's maximum jump. Participants then performed a two‐legged jump, touched the mark representing 50% of their maximum jump with one hand, and landed on the force plate with their dominant leg. Upon landing, they placed their hands on their thighs, raised their heads, looked straight ahead, and maintained this position for 20 s [18]. It was important for them to avoid falling or touching the ground with the other hand or foot, and to jump to only 50% of their maximum height. The time taken to achieve stability as TTS and peak vertical GRF in this position was then calculated. The TTS is the time it takes for an individual to return to a baseline or stable state following a jump or landing and is an indicator of dynamic stability. Total TTS is equal to the root of the sum of the squares in the medial‐lateral and anterior‐posterior directions

$$\text{RRVTTS}=\sqrt{{\mathrm{MLTTS}}^{2}+{\mathrm{APTTS}}^{2}}.$$

When analyzing COP variables during single‐ and two‐legged standing, we considered it as static balance. Additionally, we regarded F0 and F1 loading rates during walking, as well as TTS and peak vertical GRF during jumping‐landing test, as indicators of dynamic balance.

After measuring the balance outcomes in the laboratory, the physiotherapist taught the participants of the specific group a series of exercises in a progressive manner, so they could perform them at home. The examiner stayed in touch with them over the phone to monitor their progress with the exercises. For transverse abdominis muscle strengthening, participants were instructed to slowly contract their abdominal muscles inward, making sure the movement was not sudden or intense, as it could cause other abdominal muscles to contract. They were advised to feel the contraction in the anterior superior iliac spine and hold it for 10 s. In the first week, the contraction was performed in a supine position with knees bent at 90 degrees and feet placed on the ground. In the second week, it was done on all fours. In the third week, while in the all fours position, participants raised the opposite arm and leg while contracting their abdominal. In the fourth week, they raised the opposite arm and leg with a 1 kg weight. To strengthen the thigh extensor muscles, participants pressed a ball or pillow between their knees while performing bridging in the first week. In the second week, participants performed a single‐leg bridge with one foot on the ground and raised the other leg. In the third week, they did a one‐leg bridge with a 1 kg weight, and in the fourth week, they used a 1.5 kg weight. To strengthen the thigh abductor muscles, participants lay on their side and raised their leg in the extension position without significant rotation. The bottom leg remained flexed without hip flexion or trunk movement. The resistance on the muscles was gradually increased over the weeks—the exercise was performed without weights in the first week, with a 1 kg weight in the second week, with a 2 kg weight in the third week, and with a 3 kg weight in the fourth week [19]. The thigh and abdominal muscle strengthening exercises were done in 3 sets, with 10 repetitions in each set.

Both groups of people were asked to place the LWI in a comfortable shoe and use it while standing and walking. They were also instructed to perform the exercises 3–4 times a day, make notes on the provided form, and submit it to the examiner at the end of the study. Finally, balance tests were conducted after 4 weeks using the interventions in the biomechanics laboratory.

### 2.3 Statistical Analysis

The data were statistically analyzed using SPSS software for Windows version 21 with a significance level of *p* < 0.05. The normality of the data was checked using the Shapiro–Wilk test. To compare the results before and after within each group, the paired *t*‐test was used for normally distributed data, and the non‐parametric Wilcoxon test was used for non‐normally distributed data. To compare the results between groups before and after the intervention, the independent *t*‐test was used for normally distributed data, and the Mann–Whitney test was used for non‐normally distributed data.

## 3 Results

**Figure 1:**
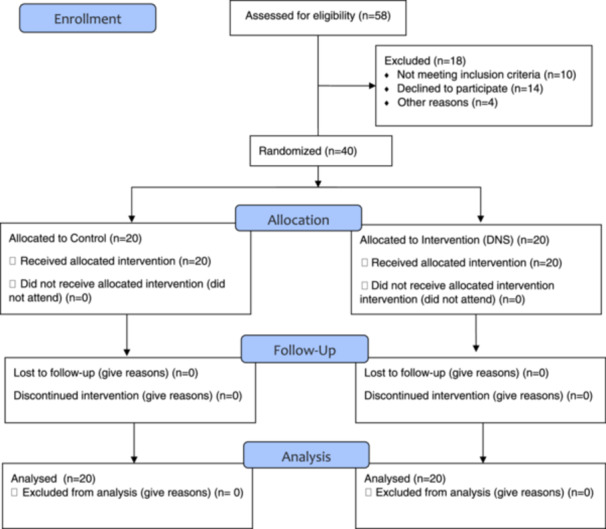
CONSORT flowchart.

All 20 subjects in each group completed the study and were analyzed (Figure 1). The demographic characteristics of the subjects are shown in Table 1, and there were no significant differences between the two groups (*p* < 0.05). The normality results revealed that only three variables—COP displacement in the anterior‐posterior direction and total displacement in two‐leg standing, and peak vertical GRF during jumping‐landing test—were normal in both groups. For the other variables, the data distribution was not normal in at least one of the test conditions.

**Table 1:** Comparing the demographic characteristics of the two groups, mean (SD).

Variables	LWI group (*n* = 20)	LWI + exercise group (*n* = 20)	*p*‐value
Quantitative variables [mean (SD)]			
Age (year)	24.40 (6.01)	29.90 (7.25)	0.053
Height (cm)	175.60 (11.14)	172.85 (8.07)	0.378
Weight (kg)	66.35 (9.39)	72.20 (11.09)	0.080
BMI (Kg/m^2^)	21.57 (3.02)	24.08 (2.80)	0.060
Qualitative variables [number (percent)]			
Gender			
Female	6 (30)	8 (40)	0.741
Men	14 (70)	12 (60)	
Dominant foot			
Right	16 (80)	14 (70)	0.716
Left	4 (20)	6 (30)	

Abbreviations: BMI, body mass index; LWI, lateral wedge insole; SD, standard deviation.

There was no statistically significant difference between the two groups before the interventions (*p* > 0.05), except for the peak vertical GRF at the TTS test (*p* = 0.012). Therefore, to account for the group differences before the intervention in this variable, an analysis of covariance (ANCOVA) was conducted.

### 3.1 Post‐Intervention Comparison Between Groups

The results of parameters related to the dynamic balance are shown in Table 2. It was found that the F1 loading rate in the LWI + exercise group was significantly lower compared to the LWI group alone (*p* = 0.01). There was no statistically significant difference between the two groups for F0 loading rate during walking (*p* = 0.63). Additionally, there were no significant differences found between the two groups for overall (*p* = 0.15), vertical (*p* = 0.15), and anterior‐posterior (*p* = 0.72) TTS, as well as peak vertical GRF (*p* = 0.16) during the jumping‐landing test.

**Table 2:** Mean, standard deviation, and significance level of the variables related to dynamic balance in the within‐group and between‐group comparisons.

	LWI group (*n* = 20)			LWI + exercise group (*n* = 20)			Comparison between groups	
Variables	Pre‐intervention, mean (SD)	Post‐ intervention, mean (SD)	*p*‐value (within‐group comparison)	Pre‐ intervention, mean (SD)	Post‐ intervention, mean (SD)	*p*‐value (within group comparison)	*p*‐value (pre‐test between group comparison)	*p*‐value (post‐test between group comparison)
Loading rate F0	0.08 (0.02)	0.13 (0.03)	< 0.001**	0.09 (0.04)	0.14 (0.04)	0.002*	0.33	0.63
Loading rate F1	0.03 (0.006)	0.04 (0.007)	0.011*	0.038 (0.008)	0.037 (0.003)	0.970	0.8	0.010*
Overall TTS	4.31 (0.99)	4.12 (0.78)	0.34	3.70 (0.41)	4.47 (1.21)	0.002*	0.07	0.15
TTS in vertical direction	4.31 (1.00)	4.12 (0.78)	0.34	3.69 (0.41)	4.48 (1.21)	0.002*	0.07	0.15
TTS in anterior‐posterior direction	3.98 (0.86)	3.90 (1.10)	0.34	3.49 (0.30)	3.69 (0.52)	0.08	0.073	0.72
VGRF in jumping‐landing test	2.41 (0.35)	2.18 (0.31)	0.013*	2.15 (0.24)	1.93 (0.24)	< 0.001**	0.012	0.16

Abbreviations: LWI, lateral wedge insole; SD, standard deviation; TTS, time to reach stability; VGRF, vertical ground reaction force.

The results of parameters related to the static balance are shown in Table 3. When comparing single‐leg standing balance, the group that used LWI along with exercise showed significantly lower COP parameters compared to the group that used LWI alone. Specifically, the standard deviation (SD) of velocity in the medial‐lateral (*p* = 0.04) and anterior‐posterior (*p* = 0.04) directions, SD of displacement in the anterior‐posterior direction (*p* = 0.01), average velocity in the anterior‐posterior direction (*p* = 0.04), and overall velocity (*p* = 0.04) were all significantly lower in the LWI with exercise group. However, there was no significant difference between the two groups when it came to COP parameters during two‐leg standing (*p* < 0.05).

**Table 3:** Mean, standard deviation, and significance level of the variables related to static balance in the within‐group and between‐group comparisons.

	LWI group (*n* = 20)			LWI + exercise group (*n* = 20)			Comparison between groups	
Variables	Pre‐intervention, mean (SD)	Post‐ intervention, mean (SD)	*p*‐value (within group comparison)	Pre‐ intervention, mean (SD)	Post‐ intervention, mean (SD)	*p*‐value (within group comparison)	*p*‐value (pre‐test between group comparison)	*p*‐value (post‐test between group comparison)
SD of velocity in AP direction in single‐leg standing	54.62 (45.10)	37.63 (20.22)	0.2	50.49 (26.03)	26.32 (11.12)	< 0.001**	0.73	0.042*
SD of velocity in AP direction in two‐leg standing	15.43 (6.54)	27.22 (17.47)	0.011*	16.85 (14.02)	33.74 (72.81)	0.117	0.69	0.13
SD of velocity in ML direction in single‐leg standing	46.32 (20.00)	26.81 (13.91)	0.004*	37.51 (13.03)	17.79 (13.19)	< 0.001**	0.107	0.05*
SD of velocity in ML direction in two‐leg standing	15.93 (13.16)	30.10 (19.46)	0.007*	18.30 (23.13)	58.73 (150.29)	0.023*	0.7	0.54
SD of displacement in AP direction in single‐leg standing	10.87 (3.77)	9.53 (2.32)	0.26	11.20 (3.00)	8.08 (2.36)	0.003*	0.76	0.015*
SD of displacement in AP direction in two‐leg standing	7.20 (2.71)	8.12 (2.80)	0.12	8.31 (5.99)	7.62 (4.61)	0.32	0.46	0.23
SD of displacement in ML direction in single‐leg standing	8.49 (2.39)	5.31 (2.64)	0.004*	8.08 (2.93)	4.81 (3.78)	0.011*	0.63	0.4
SD of displacement in ML direction in two‐leg standing	5.79 (3.80)	8.14 (4.26)	0.02*	7.40 (7.51)	7.80 (7.48)	0.3	0.4	0.16
Average velocity in the AP direction in single‐leg standing	67.00 (31.83)	44.05 (12.23)	0.02*	61.42 (20.45)	35.77 (15.08)	< 0.001**	0.52	0.05*
Average velocity in the AP direction in two‐leg standing	21.08 (7.68)	27.00 (10.72)	0.02*	23.71 (20.96)	27.29 (33.09)	0.08	0.60	0.08
Average velocity in the ML direction in single‐leg standing	61.23 (20.67)	34.25 (20.44)	0.002*	50.61 (15.06)	24.04 (19.62)	0.001*	0.08	0.12
Average velocity in the ML direction in two‐leg standing	17.60 (7.72)	24.92 (11.07)	0.02*	26.11 (37.86)	40.68 (81.02)	0.02*	0.34	0.65
Average overall velocity in single‐leg standing	101.31 (37.74)	62.77 (23.02)	0.002*	88.80 (25.86)	47.90 (26.04)	< 0.001**	0.23	0.05*
Average overall velocity in two‐leg standing	30.80 (11.82)	41.45 (17.05)	0.02*	39.79 (46.26)	54.95 (94.31)	0.033*	0.4	0.24
Average overall displacement in single‐leg standing	61.96 (30.57)	66.21 (25.30)	0.42	75.97 (27.64)	83.40 (34.88)	0.58	0.14	0.09
Average overall displacement in two‐leg standing	68.56 (28.03)	57.63 (18.24)	0.17	71.67 (19.64)	73.88 (14.71)	0.66	0.69	0.002*
Average displacement in AP direction in single‐leg standing	51.27 (27.27)	50.66 (21.78)	0.94	56.63 (24.65)	67.18 (32.46)	0.4	0.52	0.11
Average displacement in AP direction in two‐leg standing	66.71 (29.52)	54.51 (21.05)	0.17	68.83 (19.96)	68.76 (13.93)	0.97	0.8	0.021*
Average displacement in ML direction in single‐leg standing	27.78 (21.81)	35.87 (19.20)	0.13	40.90 (29.28)	42.40 (23.67)	0.63	0.12	0.67
Average displacement in ML direction in two‐leg standing	9.96 (4.88)	10.90 (5.96)	0.69	15.04 (7.22)	18.75 (11.70)	0.28	0.07	0.003*
CEA at single‐leg standing	0.07 (0.05)	0.04 (0.03)	0.12	0.07 (0.04)	0.13 (0.22)	0.46	0.72	0.81
CEA at two‐leg standing	0.03 (0.04)	0.07 (0.06)	0.02*	0.07 (0.14)	0.04 (0.03)	0.55	0.22	0.1

Abbreviations: AP, anterior‐posterior; CEA, confidence ellipse area; LWI, lateral wedge insole; ML, medial‐posterior; SD, standard deviation.

### 3.2 Pre‐Post Comparison Within Groups

For dynamic balance (Table 2), the F0 loading rate in both groups and F1 in the LWI group were significantly lower compared to pre‐intervention (*p* < 0.05). Additionally, in both groups, the peak vertical GRF was significantly decreased after the intervention compared to pre‐intervention during the jumping‐landing test (*p* < 0.05).

In single‐leg standing, the COP parameters for static balance (Table 3) showed significantly lower values for both groups compared to before the intervention (*p* < 0.05). This includes the SD of velocity and displacement in the medial‐lateral direction, average velocity in the anterior‐posterior and medial‐lateral directions, and average overall velocity. Additionally, the SD of displacement in the anterior‐posterior direction was significantly reduced in the LWI + exercise group after the intervention (*p* = 0.003), but not in the LWI group (*p* = 0.26) compared to before the intervention.

## 4 Discussion

In the post‐intervention comparison between groups, it was found that the F1 loading rate in the LWI + exercise group was significantly lower compared to the LWI group alone. When the loading rate is reduced, it means that the force's speed impacting the body is decreased. This implies that the force was applied over a longer period of time, which is seen as a positive factor [20]. The study conducted by Jafarnezhadgero et al. (2017) showed that strengthening the thigh abductor muscles in people with genuvarum reduced the vertical GRF, particularly in the medial‐lateral direction. Therefore, strengthening these muscles can help manage the factors associated with the GRF and loading rate [21]. Additionally, the thigh muscles, especially the gluteus maximus, contract during the loading response phase [11]. Consequently, based on the between‐group comparison, it appears that therapeutic exercise has an extra effect on reducing the F1 loading rate during the loading response phase compared to LWI. Strengthening these muscles enables a person to land with better control and at a lower speed. On the other hand, in the within‐group comparison, F0 loading rate in both groups and F1 in the LWI group were significantly lower compared to pre‐intervention. Even though our study's hypothesis suggested that using LWI would decrease the loading rate [22], the results did not support this. Previous studies have indicated that using an LWI reduced the loading rate and GRF in individuals with knee osteoarthritis [23, 24], which is not consistent with the results of our study. One of the reasons for this is the presence of various types of foot deformities, such as pronation, which was not part of the exclusion criteria for this study. These deformities can affect the loading rate at initial contact with the ground, and these factors varied among the individuals. Additionally, ankle stability and muscle strength can also play a significant role in reducing the loading rate, but these were not taken into account in the current study.

In the post‐intervention comparison after the intervention, COP parameters, including the SD of velocity in the medial‐lateral and anterior‐posterior directions, SD of displacement in the anterior‐posterior direction, average velocity in the anterior‐posterior direction, and overall velocity in single‐leg standing in the group using LWI along with exercise, were significantly less than in the group using LWI alone. A lower COP velocity and displacement indicate better balance and greater stability [9]. Evidence suggests that strengthening the thigh muscles can improve function, pain, and balance in individuals with knee osteoarthritis [12, 13, 25] and patellofemoral pain syndrome [26, 27]. In the context of genuvarum, Ghasemi et al. (2018) demonstrated that 12 weeks of strengthening exercises targeting the lateral thigh muscles with a theraband improved static balance in the one‐leg standing in patients with genuvarum [28], which aligns with the results of our study. Therefore, strengthening these muscles can enhance single‐leg balance compared to using LWI alone in individuals with genuvarum. Furthermore, in the within‐group comparison, a significant decrease in these variables was observed in both groups, indicating the positive effects of both interventions (LWI with and without exercise therapy). However, the combination of the two interventions had a greater effect on static balance. Several studies have explored the improvement of balance using LWI by laterally shifting the COP and providing sensory feedback [9], which is consistent with the findings of this research. In the realm of static balance, a study by Esfandiari et al. [29] indicated the positive impact of LWI on static balance during single‐leg standing using a force plate in individuals with knee osteoarthritis. The distinction between these studies and our present study is that we evaluated individuals with genuvarum without knee osteoarthritis.

The comparison of both groups in terms of COP during two‐leg standing showed a significant increasing trend, which is not a positive sign for balance improvement. In single‐leg standing, more activity of the thigh muscles, especially the gluteus medius and abdomen, is required for body stability. In comfortable two‐leg standing, thigh muscles such as gluteus maximus and medius are less active. Strengthening these muscles seems to improve stability in single‐leg standing for the group receiving exercise therapy [11, 30]. The increase in COP could be due to not using the LWI during the laboratory test, which might have increased balance stability in two‐leg standing. In a study by Zangi et al. (2017), using LWI reduced COP velocity and foot displacement in the medial‐lateral direction during two‐leg standing, which differs from our study's results [9]. In Zangi's study, shoes and LWI were used during the test, and the effect of shoes was not excluded, while our study evaluated people in bare feet. Additionally, participants in the previous study were also using medication, so pain reduction in people with osteoarthritis using medication may have contributed to balance improvement.

In both groups, the intervention led to a significant decrease in vertical GRF compared to pre‐intervention. Previous studies have also reported a decrease in vertical GRF when using a LWI in individuals with knee osteoarthritis while running, which aligns with the results of our study. Ueno et al. [31] found that weak thigh abductor muscles and poor pelvic floor control increased vertical GRF during jumping‐landing test. Therefore, exercises focusing on thigh control and hip motion may help in controlling vertical GRF. Esculiar et al. [32] also demonstrated the positive impact of thigh abductor strengthening exercises on reducing vertical GRF during running in individuals with patellofemoral pain syndrome. However, in our study, there was no significant difference between the two groups after the interventions, indicating that therapeutic exercise did not have an additional effect on the use of lateral wedge in reducing this variable. This disparity between our study and previous research may be attributed to differences in participant characteristics, types of exercises, and intervention durations, rather than the use of LWI during the balance test in previous studies.

The following limitations should be taken note in this study:

– The assessment was limited to young people and may not be applicable to the entire population.
– The task of jumping and landing on one leg was performed at a single height, limiting variability.
– Only a 5 mm leg‐heel width index (LWI) was used; other degrees were not considered.
– Testing was conducted barefoot due to restrictions on using shoes and LWI on the force plate.
– The study did not evaluate individuals with severe deformities, as most participants had similar genuvarum degrees.
– The study did not evaluate impact of shoes and participants did not wear the same shoes wearing the LWI

For future studies, the following are recommended:

– Investigate the effects of thigh and abdominal exercises on lower limb alignment.
– Conduct studies across a wider age range to prevent complications associated with deformities.
– Examine the long‐term effects of interventions.
– Evaluate the electromyographic activity of lower trunk muscles.

## 5 Conclusion

Based on the findings, both the use of a LWI alone or in combination with therapeutic exercise had a positive impact on both static balance during single‐leg standing and dynamic balance measured by vertical GRF in the jumping‐landing test. Additionally, our study revealed that strengthening the thigh and abdominal muscles could further enhance the improvement in static balance during single‐leg standing and dynamic balance using loading rate during walking when combined with LWI. Therefore, incorporating muscle strengthening exercises targeting the muscles around the knee, in addition to using LWI, can be beneficial in the rehabilitation of individuals with genuvarum, helping to prevent injuries related to poor posture control.

## Author Contributions

**Nafiseh Shahri:** methodology, investigation, writing–original draft. **Aliyeh Daryabor:** writing–review and editing, conceptualization, supervision. **Mehdi Rezaei:** formal analysis, investigation. **Abbas Rahimi:** conceptualization, data curation, supervision, writing–original draft.

## Ethics Statement

The current study was a randomized clinical trial, registered in the Iranian registry of clinical trials (registration number: IRCT20221103056387N1), and the Ethics committee of Shahid Beheshti University of Medical Sciences approved all protocols (IR.SBMU.RETECH.REC.1401.149).

## Consent

Written informed consent or assent (as appropriate) was obtained from all participants and their legal guardians.

## Conflicts of Interest

The authors declare no conflicts of interest.

## Transparency Statement

The lead author Aliyeh Daryabor affirms that this manuscript is an honest, accurate, and transparent account of the study being reported; that no important aspects of the study have been omitted; and that any discrepancies from the study as planned (and, if relevant, registered) have been explained.

## Data Availability

The data that support the findings of this study are available on request from the corresponding author. The authors have nothing to report.
